# Extractions, Contents, Antioxidant Activities and Compositions of Free and Bound Phenols from Kidney Bean Seeds Represented by ‘Yikeshu’ Cultivar in Cold Region

**DOI:** 10.3390/foods13111704

**Published:** 2024-05-29

**Authors:** Lei Zhu, Chuan Zhan, Xinchu Yu, Xixi Hu, Sibo Gao, Yanqing Zang, Di Yao, Changyuan Wang, Jingyu Xu

**Affiliations:** 1 College of Food Science and Technology, Heilongjiang Bayi Agricultural University, Daqing 163319, China; zhulei2580@126.com (L.Z.); zc021212@163.com (C.Z.); 13946955388@163.com (X.Y.); 13836746105@163.com (S.G.); byndzangyanqing@163.com (Y.Z.); yd13845991700@163.com (D.Y.); 2 National Coarse Cereals Engineering Research Center, Daqing 163319, China; 3 Agri-Food Processing and Engineering Technology Research Center, Daqing 163319, China; 4 Daqing Branch, Heilongjiang Academy of Agricultural Sciences, Daqing 163319, China; huxixi116@163.com; 5 College of Agriculture, Heilongjiang Bayi Agricultural University, Daqing 163319, China

**Keywords:** kidney bean, free and bound phenols, content, antioxidant activities, composition, extraction, seed coat and cotyledon

## Abstract

To thoroughly understand the profile of phenolic phytochemicals in kidney bean seeds cultivated in a cold region, the extractions, contents, antioxidant activities, compositions of free and bound phenols in the seed coat and cotyledon, and also relevant color attributes, were investigated. The results indicated that ultrasound-assisted extraction was an efficient method for free phenols. The bound phenols in seed coat and cotyledon were released more efficiently by alkali-acid and acid-alkali sequential hydrolysis, respectively. Under the optimized extractions, total phenols (TPC), flavonoids (TFC), and anthocyanins (TAC) ranged in 7.81–32.89 mg GAE/g dw, 3.23–15.65 mg RE/g dw, and 0–0.21 mg CE/g dw in the whole seeds of the five common kidney beans. There was a big difference in phenolic distribution between red and white seeds. From whole seed, the phenols in the four red cultivars mainly existed in free state (78.84%) and seed coat (71.56%), while the phenols in the white ‘Sark’ divided equally between free (51.18%) and bound (48.82%) states and consisted chiefly in cotyledon (81.58%). The correlation analyses showed that the antioxidant activities were significantly and positively correlated with TPC and TFC. The phenolic attributes were closely associated with the color of the seed coat. Red seeds had higher total contents of phenols than white seeds. TAC had a positively significant correlation with redness. Brightness and yellowness showed a negatively significant correlation with TPC, TFC, and antioxidant capacities, which were necessarily linked with redness degree and spot in red seeds. The spotted red ‘Yikeshu’ with the most outstanding performance on phenolic attributes was selected to analyze phenolic compounds with UHPLC-QE-MS. Among the 85 identified phenolics, 2 phenolic acids and 10 flavonoids were dominant. The characteristic phenolics in free and bound states were screened in both seed coat and cotyledon, respectively. The available information on the phenolic profile may expand the utilization of kidney beans as a nutritional ingredient in the food industry.

## 1. Introduction

Kidney bean (*Phaseolus vulgaris* L.), as an important kind of legume, is cultivated more widely than other legumes around the world [1]. It is also called navy bean, red bean, black bean, pinto bean, or cranberry bean [2]. In China, kidney beans are one of the essential ingredients in a daily diet. Heilongjiang Province, located in the extreme northeast of China, is one of the main production areas for kidney beans of high edible quality. In recent years, the planting area and export volume of kidney beans in Heilongjiang Province have gradually increased. The main cultivars, such as ‘En’, ‘Zihua’, ‘Sark’, ‘Japanese red’, ‘Spain White’, and ‘British red’, belong to the types of milk-speckled, purple-speckled, white, and red kidney beans in Heilongjiang province [3].

The kidney beans are rich in nutrients such as protein, dietary fiber, carbohydrates, and mineral elements such as calcium, iron, potassium, and sodium [2]. Furthermore, a large number of bioactive substances exist in kidney beans, including enzyme inhibitors, lectins, and phenols [4], attracting more and more attention. Among them, phenols have been proven to have many biological activities, such as antioxidant, obesity prevention, anticancer, lowering blood glucose levels, improving inflammation, etc. [5,6,7,8,9], which makes them a wide prospect in foods, pharmaceuticals ,and cosmetics [10,11,12].

Their molecules consist of one or more aromatic rings that contain one or more hydroxyl substituents. In addition, they are divided into flavonoids and non-flavonoids. Flavonoids have a C15 (C6-C3-C6) general structural backbone [13], including flavonols, flavones, flavanols, flavanones, isoflavones, and anthocyanins. Phenolic acids are the most common and main type of non-flavonoids, including hydroxybenzoic acids and hydroxycinnamic acids [14]. The seeds of kidney beans have a relatively low phenol content, but they are widely consumed in the daily diet. Therefore, the analysis of the phenol content and composition has a significant value in the development and use of kidney bean seeds and their phenolic ingredients. Phenols exist mainly in free or bound states in plants [15]. Free phenols are monophenols with one or more hydroxyl groups in their molecules, which can be extracted by organic solvents and also assisted by microwaves or ultrasounds [16]. However, for bound phenols, the ester and glycosidic bonds between phenols and cell wall polymers cannot be released by organic solvents, which can be hydrolyzed from complexes by alkali, acids, and enzymes [17]. There is a significant variation in the phenol content among different beans. According to previous reports, total phenol content ranged from 0.11 to 4.59 mg GAE/g dw in kidney beans [18,19], 1.70 to 3.43 mg GAE/g dw in mung beans [20,21], and 0.66 to 2.66 mg GAE/g dw in peas [22]. Phenolic compounds were also identified and determined in different beans in many studies. Common flavonoids include quercetin, naringenin, rutin, catechin, and luteolin, and the main phenolic acids contain caffeic acid, chlorogenic acid, coumaric acid, ferulic acid, and gallic acid [23,24].

The phenolic profile of kidney beans cultivated in cold regions has been lacking an indepth study. Therefore, in this research, the extractions of free and bound phenols in seed coat and cotyledon were first optimized, respectively. Subsequently, the total contents and antioxidant activities of phenols as well as related color attributes of five common kidney beans collected from the cold region were systematically investigated. In particular, the phenolic compounds in ‘Yikeshu’ seed with high total content were comprehensively analyzed. This study can provide fundamental data for the application of kidney bean seeds in the food industry and further research into their phenolic compounds.

## 2. Materials and Methods

### 2.1. Samples and Reagents

The seeds of five kidney bean cultivars, including ‘British red’, ‘Japanese red’, ‘Yikeshu’, ‘Zhuhong’, and ‘Sark’, were collected from the experimental cultivation base of the National Coarse Cereals Engineering Research Center in Daqing City, Heilongjiang Province, China. The represented variety ‘Yikeshu’ was used for optimization of phenolic extraction and all the determination, and the other four cultivars were applied together to compare and analyze the basic nutritional components, color traits, total contents, and antioxidant activities of phenols from seeds.

The 1,1-diphenyl-2-trinitrophenylhydrazine (DPPH) (≥98%), 2,2′-Azinobis (3-ethylbenzothiazoline-6-sulfonic Acid) Ammonium Salt (ABTS) (≥98%), 2,4,6-Tri(2-pyridyl)-1,3,5-triazine (TPTZ) (≥98%), and 6-hydroxy-2,5,7,8-tetramethylchroman-2-carboxylic acid (Trolox) (≥98%) were purchased from Yuanye Bio-Technology Co., Ltd. (Shanghai, China). The standards, gallic acid (≥98%) and rutin (≥98%), were obtained from Sigma-Aldrich (St. Louis, MO, USA). The mass spectrum grade reagents, ammonium acetate, methanol, acetonitrile, and acetic acid, were purchased from Aladdin Bio-Chem Technology Co., Ltd. (Shanghai, China), Anpel Laboratory Technologies Inc. (Shanghai, China), and Thermo Fisher Scientific Inc. (Waltham, MA, USA), respectively. All analytical grade chemicals and reagents were purchased from Aladdin Bio-Chem Technology Co., Ltd. (Shanghai, China).

### 2.2. Chromatic Determination

Chromatic feature was determined on kidney seed using the CIE L*a*b* system and a colorimeter (CS-580A, Hangzhou Caipu Technology Co., Ltd., Hangzhou, China) with illuminant D65 and observer, angle of 10°. A total of 50 seeds randomly selected from each variety were placed one by one in a Petri dish to determine the chromatic indicators. In addition, the L*, a*, and b* values measured were inputted into the Colortell color tool (https://www.colortell.com/ accessed on 15 August 2023) to get the converted fitting graph [25]. The chroma (C*) and hue (H*) were calculated using the following formulas:

(1) $$C*={\left[{\left(a*\right)}^{2}+{\left(b*\right)}^{2}\right]}^{\frac{1}{2}}$$

(2) $${H*=\tan}^{-1}\left(\frac{b*}{a*}\right)$$

### 2.3. Pretreatments of Kidney Seeds

The kidney bean seeds were soaked with purified water, and then the seed coat was separated from the cotyledon by tweezers. The wet materials were dried at 43 °C for 12 h in an air blast drying oven (BJPX-HGZ, Boke Scientific Instrument Co., Ltd., Jinan, China). The dried samples were then pulverized with a stainless-steel grinder (JYS-M01; Joyoung Co., Ltd., Jinan, China), and stored in vacuum packaged polyethylene pouches at −80 °C before further study [26].

### 2.4. Extraction of Free Phenols

The test aiming to optimize the extraction conditions was performed on the ‘Yikeshu’ cultivar. The free phenols were obtained by ultrasonic-assisted extraction with an ethanol-water solution. To optimize the conditions, Box-Behnken designs were utilized for the cotyledon (Table S1) based on single-factor experiments (Figure S1). In previous research, we obtained the optimal conditions for extracting free phenols from the seed coat of ‘Yikeshu’, including an ethanol volume fraction of 42%, a solid-liquid ratio of 1:42, and an ultrasonic power of 308 W, 51 min, and 4 times [27]. Therefore, these conditions were directly used to extract the free phenols from the seed coat of kidney beans in this study. Briefly, a gram of seed coat/cotyledon powder was weighed and extracted four times. The supernatants collected each time after centrifugation were concentrated at 40 mL by rotary evaporation (RE-52A, Shanghai Aige Scientific Instrument Co., Ltd., Shanghai, China).

### 2.5. Extraction of Bound Phenols

The bound phenols in ‘Yikeshu’ seed by acid and alkaline hydrolysis were released from the resulting residue after the extraction of free phenols, according to Peng et al. [26], with a small modification. The processes included acid hydrolysis, acid-alkali sequential hydrolysis, alkaline hydrolysis, and alkali-acid sequential hydrolysis.

#### 2.5.1. Acid Hydrolysis and Acid-Alkali Sequential Hydrolysis Treatment

The extraction residue of free phenols was hydrolyzed with 2 M HCl (25 mL) for 1 h at 85 °C. The solution was then adjusted to pH 2 with 6 M NaOH and centrifuged (TD5A-WS, Hunan Xiangyi Laboratory Instrument Development Co., Ltd., Changsha, China) to separate the supernatant, which was the acid hydrolysate. Subsequently, the residue of acid hydrolysis was adjusted to neutral and hydrolyzed with 2 M NaOH (25 mL) for 4 h at room temperature. Then the solution was adjusted to pH 2 with 6 M HCl and centrifuged to separate the supernatant, which was the alkali-after-acid hydrolysate. The bound phenols released in both steps of hydrolysate were extracted with ethyl acetate, respectively. The volume of ethyl acetate was the same as that of the hydrolysate supernatant. This extraction was repeated three times. The organic phases were collected, and the mixture was subjected to rotary evaporation to dryness. The dried extracts were redissolved with ethonal to 10 mL [26].

#### 2.5.2. Alkaline Hydrolysis and Alkali-Acid Sequential Hydrolysis Treatment

The extraction residue of free phenols was hydrolyzed with 2 M NaOH (25 mL) for 4 h at room temperature. The solution was then adjusted to pH 2 with 6 M HCl and centrifuged to separate the supernatant, which was the alkaline hydrolysate. Subsequently, the residue of alkaline hydrolysis was adjusted to neutral and hydrolyzed with 2 M HCl (25 mL). The solution was then adjusted to pH 2 with 6 M NaOH and centrifuged to separate the supernatant, which was the acid-after-alkali hydrolysate. The extraction with ethyl acetate and the next sample preparation were the same as in Section 2.5.1 [26].

### 2.6. Determination of the Total Content of Phenol, Flavonoid and Anthocyanin

#### 2.6.1. The Total Phenol Content

The total phenol content (TPC) was tested using the Folin-Ciocalteu method [28]. For each test sample, 0.1 mL of extract was mixed in sequence with 2 mL Folin-Ciocalteu reagent and 2 mL of Na_2_CO_3_ (0.5 mol/L). In addition, the reaction mixture was filled with 25 mL of distilled water. Then the sample was incubated at room temperature for 60 min. The absorbance was determined at 725 nm with a T6 New Century spectrometer (Beijing Purkinje General Instrument Co., Ltd., Beijing, China). Gallic acid (0.3–15.0 mg/mL) was used as a standard compound to establish the standardization curve, and the absorbance was converted to TPC expressed as mg gallic acid equivalents (GAE)/g dried weight (dw).

#### 2.6.2. The Total Flavonoid Content

The total flavonoid content (TFC) was determined by the aluminum chloride method [29]. For each test sample, 0.5 mL of extract was mixed in sequence with 4.5 mL of 60% ethanol, 0.3 mL of 5% NaNO_2_, 0.3 mL of 10% AlCl_3,_ and 4 mL of 4% NaOH. Also the reaction mixture was filled with 60% ethanol in 10 mL. The absorbance was determined at 510 nm. Rutin (0.0125 to 0.0100 mg/mL) was used as a standard compound to establish the standardization curve, and the absorbance was converted to TFC expressed as mg rutin equivalents (RE)/g dw.

#### 2.6.3. The Total Anthocyanin Content

Total anthocyanin content (TAC) was measured according to the pH differential method [30]. The 0.8 mL extract was respectively prepared with pH 1.0 buffer (0.2 mol/L KCl:0.2 mol/L HCl:distilled water = 50:97:53) and pH 4.5 buffer (The 9.647 g sodium acetate trihydrate and 12 mL glacial acetic acid was filled with distilled water to 250 mL) and fixed to 10 mL. The absorbance was determined at 520 nm and 700 nm. The absorbance was converted to TAC and expressed as mg cyanidin-3-glucoside equivalents (CE)/g dw. The TAC was calculated using the following formula:

(3) $$\mathrm{TAC}(mg\;\mathrm{CE}/g\;\mathrm{dw})=\frac{A\times \mathrm{MW}\times \mathrm{DF}\times 10^{3}}{\varepsilon \times 1}$$

where A = (A_520nm_ − A_700nm_) pH 1.0 − (A_520nm_ − A_700nm_) pH 4.5; MW (molecular weight) = 449.2 g/mol for cyanidin-3-glucoside; DF = dilution factor established in D; l = pathlength in cm; ε = 26,900 molar extinction coefficient, in L × mol^−1^ × cm^−1^, for cyanidin-3-glucoside; and 10^3^ = factor for conversion from g to mg.

### 2.7. Determination of Antioxidant Activities

#### 2.7.1. The Ability to Eliminate DPPH Free Radicals

The DPPH assay was referred to by Pang et al. [31]. Briefly, 0.1 mL of extract was mixed with 3.9 mL of DPPH methanolic solution (0.0025 g/100 mL). The mixture was incubated in the dark for 30 min. The absorbance was measured at 515 nm. Trolox (2–10 μmol/L) was used to establish the standardization curve, and the results were converted into Trolox equivalents (µmol TE/g dw).

#### 2.7.2. The Ability to Eliminate ABTS Free Radicals

The ABTS assay referred to Hong et al. [32]. Briefly, 0.05 mL of extract was mixed with 5 mL of ABTS solution (7.4 mmol/L ABTS:2.6 mmol/L K_2_S_2_O_8_ = 1:1). The mixture was incubated at room temperature for 6 min. The absorbance was measured at 734 nm.

#### 2.7.3. The Ferric-Reducing Antioxidant Power

The ferric-reducing antioxidant power (FRAP) assay was referred to by Benzie et al. [33]. For each test sample, 0.2 mL of extract was mixed in sequence with 6 mL of FRAP solution (300 mmol/L acetate buffer; 10 mmol/L TPTZ; 20 mmol/L FeCl_3_ = 10:1:1) and 0.6 mL of distilled water. The mixture was incubated in a water bath at 37 °C for 30 min. The absorbance was measured at 596 nm.

### 2.8. Analysis of Phenolic Compounds by UHPLC-QE-MS

The extracts of free and bound phenols in the seed coat and cotyledon under optimal conditions were dried in nitrogen flow, then redissolved with a 200 μL solution of methanol and water (methanol:water = 3:1) and vortexed for 30 s. Redissolved samples were sonicated (TS-1200, Tianshi Zhongmei Technology Co., Ltd., Beijing, China) for 10 min in an ice-water bath, then centrifuged (Heraeus Fresco17, Thermo Fisher Scientific, Shanghai, China) at 12,000 rpm for 15 min at 4 °C. The resulting supernatants were transferred to glass sample vials for analysis.

Phenolic compound analyses were persuaded using an Ultimate 3000 UPLC coupled to a Q Exactive HFX mass spectrometer (Thermo Fisher Scientific, Waltham, MA, USA) with a Waters ACQUITY UPLC HSS T3 column (2.1 mm × 100 mm, 1.8 μm). The mobile phase consisted of an aqueous solution containing 5 mmol/L ammonium acetate and 5 mmol/L acetic acid (phase A) and acetonitrile (phase B). The auto-sampler temperature was 4 °C, and the injection volume was 3 μL. The gradient program was as follows: 0–0.7 min, 1% B, flow 0.35 mL/min; 0.7–9.5 min, 1–99% B, flow 0.35 mL/min; 9.5–11.8 min, 99% B, flow 0.35–0.5 mL/min; 11.8–12 min, 99–1% B, flow 0.5 mL/min; 12–14.6 min, 1% B, flow 0.5 mL/min; 14.6–14.8 min, 1% B, flow 0.5–0.35 mL/min; 14.8–15 min, 1% B, flow 0.35 mL/min. MS analysis was conducted using electrospray ionization (ESI) and a full scan MS spectrum. ESI source conditions were set as the following: sheath gas flow rate as 30 Arb, auxiliary gas flow rate as 10 Arb, capillary temperature 350 °C, full MS resolution as 60,000, MS/MS resolution as 7500, collision energy as 10/30/60 in NCE mode, spray voltage as 4.0 kV (positive) or −3.8 kV (negative), respectively.

### 2.9. Statistical Analysis

Three biological replicates were performed for each assay. The data from this study were presented as the mean ± standard deviation for each sample. One-way analysis of variance (ANOVA) followed by Duncan’s multiple range test to determine statistically different values on the level of significance at *p* < 0.05. Correlation analyses and the heatmap were done by TBtools v2.084. The Venn diagrams were drawn by Origin. Differential phenolic compounds were screened on the basis of VIP > 1, Log_2_ FC ≥ 1 (up), and Log_2_ FC ≤ −1 (down).

## 3. Results

### 3.1. Optimization of Phenolic Extraction

#### 3.1.1. Optimization of Free Phenols Extraction

**Figure 1:**
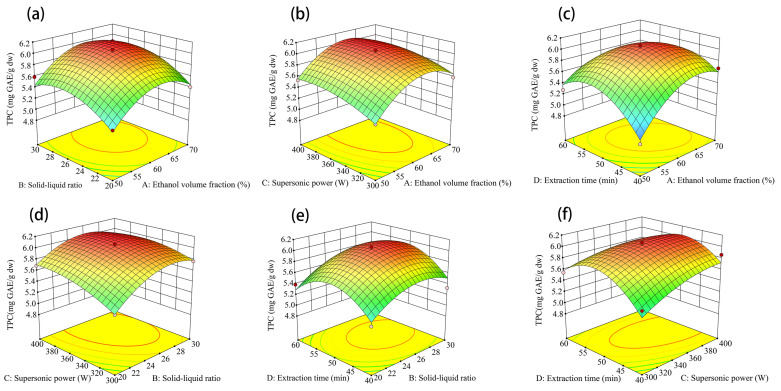
Response surface diagrams of factor interactions for the cotyledon of ‘Yikeshu’ seed. Ethanol volume fraction and solid-liquid ratio (**a**); Ethanol volume fraction and supersonic power (**b**); Ethanol volume fraction and extraction time (**c**); Solid-liquid ratio and supersonic power (**d**); Solid-liquid ratio and extraction time (**e**); Supersonic power and extraction time (**f**). Abbreviation: TPC—total phenol content.

To maximize the free phenol yield from the cotyledon of ‘Yikeshu’, ultrasonic-assisted extraction conditions were optimized by response surface methodology (RSM) based on single-factor experiments (Supplementary Figure S1). The results showed that under the conditions of ethanol volume fraction of 62%, material-liquid ratio of 1:26, ultrasonic power of 373 W, extraction time of 49 min and three times, the total phenol content of free phenol of cotyledon (CF) was the highest, which was 6.10 mg GAE/g dw (Figure 1). Meanwhile, the most effective of the free phenols extracted from seed coat (SF) had a TPC of 218.92 mg GAE/g dw [27].

Three validation tests were carried out under optimal process conditions. The mean phenols extractions were 6.23 mg GAE/g DW for CF. The relative error of the predicted values was 2.13%.

#### 3.1.2. Extraction Sequence of Bound Phenols

**Figure 2:**
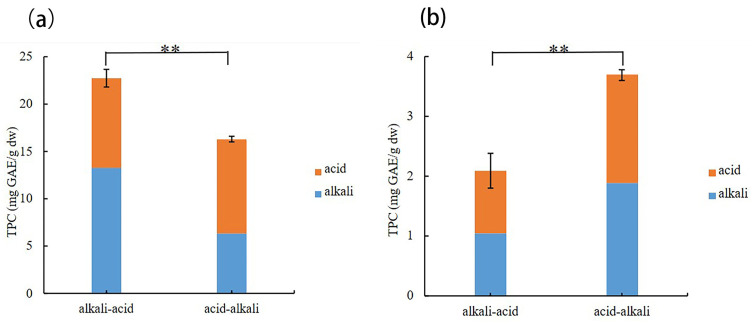
Extraction contents of bound phenols in the seed coat (**a**) and cotyledon (**b**) of ‘Yikeshu’. ** represents significant a difference at *p* < 0.01.

The residue after free phenol extraction from bean seeds contains a fair number of bound phenols. These bound phenols exist mainly in the form of glycosylation and esterification, which can be efficiently released by alkaline and acidic hydrolyses, respectively [34]. Different sequences of chemical hydrolysis were compared in the release efficiency of bound phenols in ‘Yikeshu’ seed (Figure 2). To confirm the content and distribution of phenolic compounds in different states and parts of the ‘Yikeshu’ seed, the bound phenol extracts of alkali hydrolysis (SBC) and acid-after-alkali hydrolysis (SB-AC) could be combined as the sum of alkali-acid sequential hydrolysis (SBC + SB-AC), and the bound phenol extracts of acid hydrolysis (CAC) and alkali-after-acid hydrolysis (CA-BC) could be added to the acid-alkali sequential hydrolysis (CAC + CA-BC). In general, whether for a single hydrolysate or for a sequential hydrolysate, (SBC + SB-AC) had a higher release yield with a TPC of 22.72 mg GAE/g dw for seed coat (Figure 2a), while more abundant bound phenols with a TPC of 3.69 mg GAE/g dw were found in (CAC + CA-BC) for cotyledon (Figure 2b).

### 3.2. Chromatic Feature

The color of the seeds is a crucial trait because consumers presume the quality and taste of food based on the appearance of the outside before making a decision [35]. Furthermore, the chromatic feature was closely related to the phenol content and composition of kidney bean seed. Four kidney bean cultivars, including two whole red seeds (‘British red’ and ‘Japanese red’), one spotted red seed (‘Zhuhong’), and one white seed (‘Sark’), together with the spotted red ‘Yikeshu’ common in Northeast China, were collected (Table 1). In general, ‘Sark’ seeds had higher brightness (L*, 80.50), yellowness (b*, 21.68), and lower redness (a*, 7.74) than the other four red kidney bean seeds. Among the four red seeds, ‘Zhuhong’ showed the lowest L* (34.57), ‘Japanese red’ had the lowest b* (3.61) and the highest a* (28.49) was found in ‘British red’. While there were no significant differences in L*, a*, and b* among other cultivars, respectively. The saturation of the color is expressed as chroma (C*). The C* values indicated that the color saturation of ‘British red’ was obviously higher than that of other seeds, which had no statistical difference. The hue angle (H*) is mainly used to represent hue changes in color space. The H* values represented that the ‘Sark’ seed had a yellow hue and other seeds had a red hue (Table 1). These attributes indicated that a* and H* had a similar trend, which was the direct opposite of L* and b*. Although the C* values had almost no correlation with other attributes (Figure S2).

### 3.3. Total Content and Antioxidant Activities of Phenols in Extractions

The optimal conditions described in Section 2.4 and Section 3.1 were used to extract the free and bound phenols from the seed coats and cotyledons of the five kidney beans. Then the total contents and antioxidant activities of the phenols were determined and compared.

#### 3.3.1. Total Phenol Content

**Figure 3:**
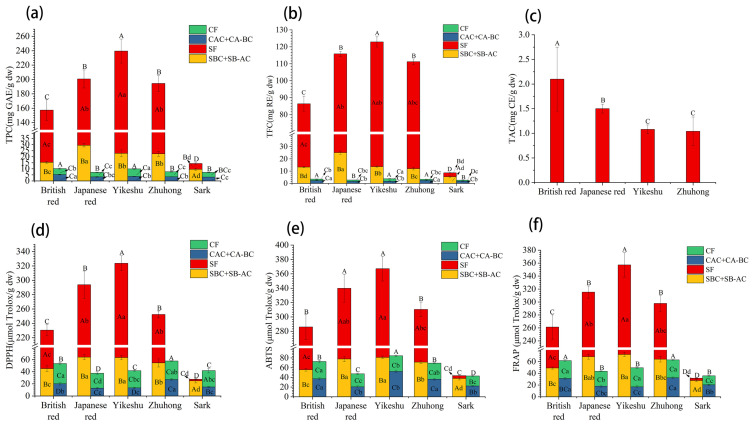
Total contents and antioxidant activities of phenols extracted from the seed coat and cotyledon of kidney beans. Total phenol contents (TPC) (**a**). Total flavonoid contents (TFC) (**b**). Total Anthocyanin Contents (TAC) (**c**). DPPH-radical scavenging activity (DPPH) (**d**). ABTS-radical scavenging activity (ABTS) (**e**). Ferric-reducing antioxidant power (FRAP) (**f**). Different capital letters in the columns of the same variety were statistically significant (*p* < 0.05) among different samples. Different lowercase letters in the columns with the same color were statistically significant (*p* < 0.05) among bean cultivars. Different capital letters on top of the columns were statistically significant (*p* < 0.05) in the total contents of the same seed part among different cultivars.

For the same kidney cultivars, the phenols in the seed coat were significantly more abundant than those in the cotyledon. But the distribution of free and bound phenols was different between red and white kidney beans (Figure 3a). The TPC in the four red kidney bean seeds decreased with the order of SF, (SBC + SB-AC), CF and (CAC + CA-BC). Furthermore, SF showed extremely high TPC (175.60 mg GAE/g dw), which was 7.83, 36.91, and 44.94 times higher than those in (SBC + SB-AC), CF and (CAC + CA-BC) on average, respectively. While in the white kidney ‘Sark’, the bound phenols extract from the seed coat (SBC + SB-AC) had the highest TPC (9.51 mg GAE/g dw), followed by SF (4.86 mg GAE/g dw) and CF (3.90 mg GAE/g dw), and the lowest was in the cotyledon bound phenol extract (CAC + CA-BC, 3.18 mg GAE/g dw) (Figure 3a).

There were significant differences in TPC between different kidney seeds. In seed coat, the white ‘Sark’ had obviously lower TPC than the red cultivars, either in free or bound phenol extracts. The ‘Yikeshu’ SF had the highest TPC (216.82 mg GAE/g dw), followed by ‘Zhuhong’ and ‘Japanese red’. While the TPC of (SBC + SB-AC) in the ‘Japanese red’ seed coat was highest (29.34 mg GAE/g dw), followed by those of ‘Yikeshu’ and ‘Zhuhong’. In cotyledon, the ‘British red’ showed the highest TPC in the CF and (CAC + CA-BC) extracts among the five kidney cultivars. According to an average weight ratio (1:9) of seed coat and cotyledon in the whole kidney beans investigated previously, the total TPC in the whole seeds varied in the order of ‘Yikeshu’ (32.89 mg GAE/g dw) > ‘Japanese red’ (26.44 mg GAE/g dw) > ‘Zhuhong’ (26.24 mg GAE/g dw) > ‘British red’ (24.83 mg GAE/g dw) > ‘Sark’ (7.81 mg GAE/g dw) (Figure 3a).

The variations of TFC were similar to TPC (Figure 3b). ‘Yikeshu’ had the highest TFC in SF (109.29 mg RE/g dw), CF (1.95 mg RE/g dw), and total (15.65 mg RE/g dw). Apparently, higher TPC and TFC were observed in spotted kidney red beans than in whole red beans in the present study. Anthocyanins were only detected in SF from the four red kidneys (Figure 3c). ‘British red’ showed the highest TAC (2.10 mg CE/g dw), followed by ‘Japanese red’ (1.50 mg CE/g dw). Both spotted red seeds, ‘Yikeshu’ and ‘Zhuhong’, possessed relatively lower TAC (1.08 and 1.04 mg CE/g dw, respectively).

There were also differences in the composition of free and bound phenols between the seed coat and cotyledon. In the seed coat per unit mass, free phenols (SF) were the main state of existence in the red cultivars (TPC, 85.38–90.52%; TFC, 78.36–89.24%), while bound phenols (SBC + SB-AC) were the main in the white variety (TPC, 66.19%; TFC, 62.53%). In cotyledon per unit mass, there was not much difference in the proportions between free phenols (CF, 54.86%) and bound phenols ((CAC + CA-BC), 45.14%) for TPC, while (CAC + CA-BC) (63.47%) were the main for TFC in the five kidneys.

#### 3.3.2. Antioxidant Activities of Phenols

The antioxidant capacities of the phenol extracts from kidney bean seeds in vitro were evaluated by three methods, including DPPH, ABTS, and FRAP (Figure 3d–f). For the four red cultivars, the seed coats showed greater antioxidant activity than the cotyledons. The antioxidant activities of SF were much higher than those of the others, followed by the bound phenol extracts from the seed coat. Moreover, the lowest antioxidant abilities were found in the free and bound phenol extracts from cotyledon, between which there were no significant differences, except for DPPH in ‘British red’ and ‘Yikeshu’. For the white ‘Sark’, (SBC + SB-AC) exhibited the highest antioxidant activities. In addition, CF and (CAC + CA-BC) had higher antioxidant capacities than SF, which was different from the trend of total phenol contents. Among the five kidney beans, the total antioxidant capacities for the whole seeds varied generally in the order of ‘Zhuhong’ > ‘British red’ > ‘Yikeshu’ > ‘Japanese red’ > ‘Sark’. In cotyledon, the highest antioxidant activities of CF were found in ‘British red’, and ‘Zhuhong’ showed the highest antioxidant abilities of the bound phenol extract.

### 3.4. Analyses of the Composition of Phenols in the ‘Yikeshu’ Seed

Because the ‘Yikeshu’ seed had the highest total phenol content among the five common kidney beans, it was used to further investigate the phenolic composition. Phenolic compounds in the free and bound states that existed in the seed coat and cotyledon were systematically analyzed by UPLC-QE-MS (Table S2).

#### 3.4.1. General Analysis of Phenolic Compounds in Different Samples

**Figure 4:**
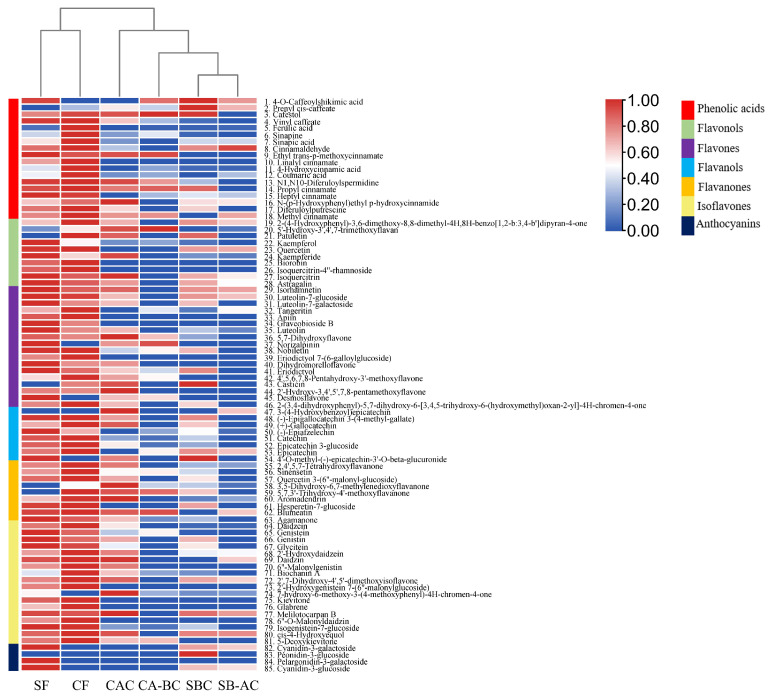
Heatmap showing phenolic compound levels in free and bound phenols extracted from the seed coat and cotyledon of ‘Yikeshu’. The intensity of color indicates phenolic compound levels using Logarithmic conversion (Log_2_) of peak area derived from UHPLC-QE-MS, and sample normalization was performed during analysis. Similarities among samples are shown above the heatmap.

A total of 85 phenolic compounds were found, including 18 phenolic acids (hydroxycinnamic acids) and 67 flavonoids (10 flavonols, 18 flavones, 8 flavanols, 9 flavanones, 18 isoflavones, and 4 anthocyanins) (Figure 4 and Table S2). Among the phenolic acids identified, hydroxycinnamic acids were the main type. According to their total levels in all six samples, 4-hydroxycinnamic acid (11) and ferulic acid (5) were dominant (Table S2). In general, CF had the highest total levels of phenolic acids, followed by SF. While the bound phenolic extracts showed relatively lower levels. Among the identified flavonoids, flavanols and isoflavones were the most abundant compounds. According to their total levels in all six samples, isogenistein 7-glucoside (79) and catechin (51) were dominant (Table S2). Anthocyanins, a kind of water-soluble flavonoids, play a vital role in the color of the plant, which exists mainly in the SF of kidney beans. Three anthocyanins derived from cyanidin, petunidin, and pelargonidin were detected in the SF samples.

**Figure 5:**
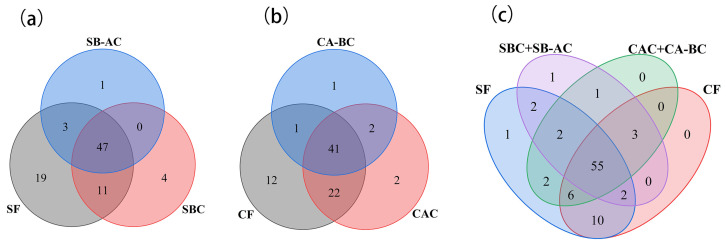
Venn diagram showing the similarities and differences of phenolic compounds detected in different samples of ‘Yikeshu’. Seed coat (**a**); Cotyledon (**b**). Free and bound phenols in the seed coat and cotyledon (**c**).

In whether seed coat (85 phenolics detected in total) or cotyledon (81 phenolics detected in total) of ‘Yikeshu’, the richness of the phenolic compounds decreased with the order of free extract (SF or CF), the first hydrolysate (SBC or CAC), and the second hydrolysate (SB-AC or CA-BC). Plus, there were more than 60 phenolic compounds detected in the extracts of both free and bound phenols (Figure 5a,b and Table S2). Among all the phenolics detected, 81 compounds existed in both seed coat and cotyledon, and 55 were common to the states of free and bound phenols. There were 10 compounds specific to free phenolics (SF and CF) compared to bound phenolics, including 1 phenolic acid (10) and 9 flavonoids (25, 26, 33, 34, 39, 73, 75, 76 and 78). Compared to phenolics in cotyledon, compound 84 was only characterized in SF, and compound 83 was only in (SBC + SB-AC). (Figure 5c and Table S2).

As shown in Figure 4, a hierarchical cluster analysis (HCA) dendrogram was obtained using all phenolic compounds detected in different states of the seed coat and cotyledon. Obviously, the 6 samples could be divided into 4 groups, which were SF, CF, CAC, and other bound phenolic extracts (CA-BC, SBC and SB-AC), respectively. The clustering result indicated that the free phenolic composition was separated from the bound phenolic composition, and the free phenolic composition in the seed coat was also distinct from that in the cotyledon, while the bound phenolic composition in CAC was different from those of the other three hydrolyzed samples.

#### 3.4.2. Screening of Characteristic Phenolic Compounds in Free or Bound States Extracted from the Seed Coat and the Cotyledon

**Figure 6:**
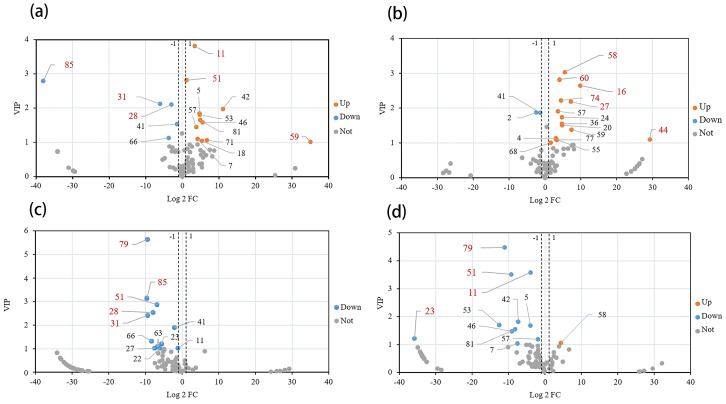
Volcano plots of differential phenolic compounds. SF vs. CF (**a**); SBC + SB-AC vs. CAC + CA-BC (**b**); SF vs. SBC + SB-AC (**c**); CF vs. CAC + CA-BC (**d**). The up-regulated (orange) and down-regulated (blue) compounds are labeled with the representative serial numbers. The red numbers also represent characteristic compounds.

To confirm the differential phenolic compounds in different states and parts of the ‘Yikeshu’ seed, differential phenolic compounds screening was performed using the 85 phenolics annotated with a criterion of |Log_2_ FC| ≥ 1 and variable importance in the project (VIP) ≥ 1 (Figure 6).

For free phenols, a total of 17 differential compounds were identified in SF vs. CF (Figure 6a). The five downregulated flavonoids (85, 31, 28, 41 and 66) were the differential phenolics of SF. The four upregulated phenolic acids (5, 7, 11 and 18) and 8 upregulated flavonoids (42, 46, 51, 53, 57, 59, 71 and 81) were the differential phenolics of CF. It is worth noting that the levels of all phenolic acids in CF were higher than in SF. For bound phenols, 17 differential compounds were found between seed coat (SBC + SB-AC) and cotyledon (CAC + CA-BC) (Figure 6b). The 1 downregulated phenolic acid (2) and 1 downregulated flavonoid (41) were the differential phenolics in (SBC + SB-AC). While the 2 upregulated phenolic acids (4 and 16) and 13 up-regulated flavonoids (20, 24, 27, 36, 44, 55, 58, 59, 60, 68,74 and 77) were the special phenolics in (CAC + CA-BC).

In the seed coat, 12 downregulated compounds, including one phenolic acid (11) and 11 flavonoids (22, 23, 27, 28, 31, 41, 51, 63, 66, 79 and 85) were screened in SF vs. (SBC + SB-AC), which were the differential phenolics of SF (Figure 6c). In cotyledon, 12 differential compounds were selected in CF vs. (CAC + CA-BC) (Figure 6d). The differential phenolics in CF contained three downregulated phenolic acids (5, 7 and 11) and eight downregulated flavonoids (23, 42, 46, 51, 53, 57, 79 and 81). Also, the differential phenolic in (CAC + CA-BC) was the one upregulated flavonoid (58).

## 4. Discussion

### 4.1. Extraction and Total Contents of Phenols in Kidney Bean Seeds

Kidney beans are well known as excellent sources of dietary fiber, protein, and carbohydrates. Moreover, the phenols in kidney bean seeds are a kind of phytochemical with high antioxidant activity. To maximize the phenol yield, the RSM was used to optimize the variables of ultrasound-assisted extraction of free phenols. The total contents of free phenols obtained under the optimal conditions ranged from 4.86 to 216.82 mg GAE/g dw of TPC, 3.25 to 109.29 mg RE/g dw of TFC, and 0 to 2.10 mg CE/g dw of TAC in the seed coat, and 3.77 to 6.23 mg GAE/g DW of TPC and 0.82 to 1.95 mg RE/g dw of TFC in the cotyledon of the five kidney beans. Sutivisedsak et al. [36] extracted free phenols with microwave assistance from eight kidney bean cultivars in North America, and the extraction of phenols ranged from 1.16 to 70.57 mg GAE/g dw in seed coats and 1.86 to 10.92 mg GAE/g dw in cotyledons, respectively. Capistrán-Carabarin et al. [37] extracted free phenols with the conventional solvent method from six cultivars of kidney beans in Oaxaca, and the extraction ranged from 81.70 to 123.30 mg GAE/g dw of TPC and 10.60 to 13.70 mg RE/g dw of TFC in seed coats and 1.81 to 2.32 mg GAE/g dw of TPC and 0.30 to 0.50 mg RE/g dw of TFC in cotyledons. In general, ultrasound-assisted extraction under the optimal conditions in this study was a highly efficient method for the free phenols of kidney bean seeds grown in cold regions.

Meanwhile, the bound phenols in the seed coat were more efficient in being released by alkali-acid sequential hydrolysis, while those in the cotyledon were more suitable for acid-alkali sequential hydrolysis for the kidney beans in this study. The bound phenol content varied from 9.52 to 29.34 mg GAE/g dw of TPC and 5.42 to 25.07 mg RE/g dw of TFC in the seed coat, and 3.69 to 5.21 mg GAE/g dw of TPC and 1.69 to 2.45 mg RE/g dw of TFC in the cotyledon of the five kidney beans. In most previous studies, alkali-acid sequential hydrolysis was selected as the most effective process for releasing the bound phenols from kidney bean seed. Chen et al. [38] and Wang et al. [39] showed that the bound phenol extractions of alkali-acid sequential hydrolysis in the whole seeds of kidney beans ranged from 0.02 to 0.12 mg GAE/g dw in Canada and 0.39 to 0.81 mg GAE/g dw in Southeast China, respectively. In the present research, it was found that the optimal sequential hydrolysis for the seed coat and cotyledon of kidney beans were different. Thus, we speculated the bound phenolic compounds existed mainly in the bound state of glycosylation in cotyledon, which can be released effectively by acidic hydrolyses, and the bound phenolic compounds, released effectively by alkaline hydrolyses, existed mainly in the esterification state in the seed coat of kidney beans.

According to the ratio of 1:9 (seed coat:cotyledon) for kidney beans, the sum phenol in the whole seeds of the five kidney beans ranged from 7.81 to 32.89 mg GAE/g dw of TPC, 3.23 to 15.65 mg RE/g dw of TFC, and 0 to 0.21 mg CE/g dw of TAC. Among them, ‘Yikeshu’ possessed the highest TPC and TFC, and ‘British red’ had the most TAC, while the lowest total phenols were found in the white ‘Sark’. According to previous research, whole mung bean seeds ranged from 1.70 to 2.50 mg GAE/g dw of total free and bound phenols [20] or 0.49 to 4.44 mg RE/g dw of total free flavonoid [21,40]. The entire seeds of the faba bean ranged from 2.58 to 5.70 mg GAE/g dw of total free phenols [41]. Among 50 beans such as pea, red bean, and soybean, the total free phenols of the seeds ranged from 0.56 to 6.98 mg GAE/g dw, and the TAC varied from 0.02 to 0.28 mg CE/g dw, respectively [42]. Therefore, the phenol content of kidney beans grown in the cold region was more abundant using optimized extraction methods compared to other beans.

It was worth noting that there was a big difference in the distribution of total phenols between the white and red kidney beans. In red kidney beans, free phenols were the primary existence state (78.84%), and phenols were distributed mainly in the seed coats (71.56%). In the white ‘Sark’, the free (51.18%) and bound phenols (48.82%) were nearly equal; the phenols consisted chiefly in the cotyledon (81.58%).

### 4.2. Correlation among Total Phenol Contents, Antioxidant Activities and Color Attributes

**Figure 7:**
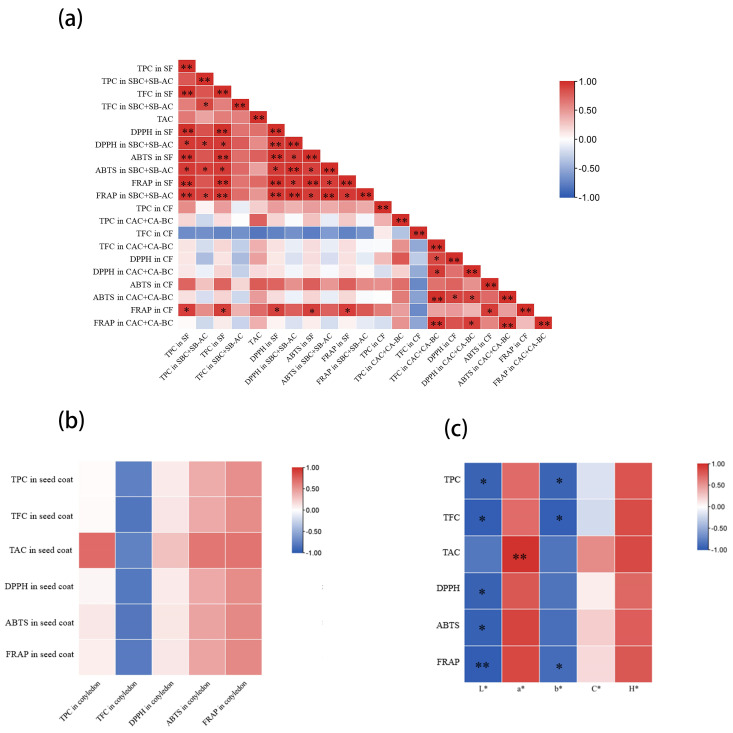
Correlations in phenol attributes among different existence states and seed parts (**a**), in sum values of phenol attributes between seed coat and cotyledon (**b**), between seed color attributes and phenol attributes of the whole seeds of in sum values of phenol attributes in seed coat and cotyledon (**c**). * represents significant correlation at *p* < 0.05, ** represents significant correlation at *p* < 0.01.

There were significantly positive pairwise correlations among TPC, TFC, DPPH, ABTS, and FRAP (*p* < 0.05. Figure 7a). Phenolic compounds are known to be very effective free radical scavengers and antioxidants [26]. There is usually a significantly positive correlation between the total content and antioxidant activity of phenols [38,43]. In this study, the more TPC and TFC the phenolic extract had, the higher DPPH, ABTS, and FRAP it exhibited. However, TAC was an exception, showing positive but not obvious correlations with other phenolic attributes (*p* > 0.05, Figure 7a). It showed that anthocyanins were the minor phenolic component in kidney seeds. We were also concerned with the connection of both seed parts and of both existence states. There were generally positive, but not significant, correlations in phenolic attributes between seed coat and cotyledon and also between free phenols and bound phenols (*p* > 0.05, Figure 7a,b).

The content of phenols can influence the color of the bean seed coat, mainly reflecting the higher level of phenols in colored beans (red or black) than in white beans [43,44]. The same result was obtained in this study. However, for red kidney beans, the degree of redness and the spots had little bearing on the total contents of phenols and flavonoids. By comparison, the spotted red seed of ‘Yikeshu’ had the highest TPC and TFC. Only TAC had a positive and significant correlation with a* (*p* < 0.01), while b* (except DPPH and ABTS) and L* showed a negative and significant correlation with other total phenol indicators of the whole seed (*p* < 0.01/0.05) (Figure 7c). These relations illustrated that redness depended on anthocyanins, and brightness and yellowness could preliminarily anticipate total phenol content (TPC and TFC) and antioxidant activity in vitro for kidney seeds.

### 4.3. Composition of Free and Bound Phenolic Compounds in the Seed Coat and Cotyledon Represented by ‘Yikeshu’

‘Yikeshu’ seeds with the highest total phenol content were chosen to analyze the phenolic composition in different extracted samples. The identified phenolic compounds contained phenolic acids and flavonoids, which are widely distributed in plants [45]. According to the total levels of the phenolic types, hydroxycinnamic acids and their derivatives were the main phenolic acids, which are the important constituents of plant cell walls [46] and play an important role in the prevention and treatment of obesity, diabetes, and associated disorders [47]. Furthermore, isoflavones, shown to prevent cancer and osteoporosis [48], were the most abundant type of flavonoids. There were 12 out of 85 compounds having more than 100 million total peak areas in the six samples, including ferulic acid (5), 4-hydroxycinnamic acid (11), quercetin (23), astragalin (28), luteolin 7-galactoside (31), eriodictyol (41), 4′,5,6,7,8-pentahydroxy-3′-methoxyflavone (42), catechin (51), epicatechin (53), isogenistein 7-glucoside (79), 5-deoxykievitone (81), and cyanidin-3-glucoside (85) (Table S2). Among them, ferulic acid was one of the main phenolic acids in kidney beans [49], quercetin derivatives were characteristic flavonols in red kidney beans [50], and catechin was dominantly phenolic in faba beans [51]. Cyanidin 3-glucoside (85) had the highest level among the four anthocyanins detected in the seed coat of ‘Yikeshu’. Cyanidin 3-galactoside (82), pelargonidin 3-galactoside (84), and cyanidin-3-glucoside (85) existed mainly in the free state, while peonidin-3-glucoside (83) had a low level only in the bound state. Chen et al. [38] found that pelargonidin 3-glucoside and cyanidin 3-glucoside only existed in the free state of kidney beans, and no anthocyanins were found in bound state. These results indicated that anthocyanins existed mainly in the free state in the seed coat of kidney beans.

Differential phenolic compounds were selected with a threshold of |Log_2_ FC| ≥ 1 and a VIP value ≥ 1 in pairwise comparison. There were significant differences in the kinds and levels of phenolics between free and bound phenols, and well as between seed coat and cotyledon. Most of the differential compounds were composed of flavonoids. To find the most representative phenolics of the differential compounds, the screening range was further narrowed by |Log_2_ FC| > 20 or VIP value > 2. The filtered compounds were identified as characteristic phenolics marked with red in pairwise comparisons (Figure 6). For free state, astragalin (28), luteolin 7-galactoside (31) and cyanidin 3-glucoside (85) were characteristic in the seed coat, and 4-hydroxycinnamic acid (11), catechin (51) and 5,7,3′-trihydroxy-4′-methoxyflavanone (59) were distinctive in cotyledon (Figure 6a). For the bound state between seed coat and cotyledon, n-(p-hydroxyphenyl) ethyl p-hydroxycinnamide (16), isoquercitrin (27), 2′-Hydroxy-3,4′,5′,7,8-pentamethoxyflavone (44), 3,5-dihydroxy-6,7-methylenedioxyflavanone (58), aromadendrin (60), and 7-hydroxy-6-methoxy-3-(4-methoxyphenyl)-4H-chromen-4-one (74) were characteristic bound phenolics in cotyledon (Figure 6b). In the seed coat, astragalin (28), luteolin 7-galactoside (31), catechin (51), isogenistein 7-glucoside (79), and cyanidin 3-glucoside (85) were the characteristic phenolics in their free state (Figure 6c). In cotyledon, 4-hydroxycinnamic acid (11), quercetin (23), catechin (51) and isogenistein 7-glucoside (79) were the characteristic phenolics in the free state (Figure 6d). The further filtration gave a clearer picture of the characteristic distribution of free and bound phenolics in the seed coat and cotyledon of kidney beans represented by ‘Yikeshu’ cultivated in a cold region, which provides a reference for the purification and bioactivity research of a certain phenolic compound.

## 5. Conclusions

A complete procedure was established to extract phenols from kidney bean seeds. The free phenols were first extracted from the seed coat and cotyledon under optimal conditions by ultrasonic assistance, respectively. Then the bound phenols in the residue of the seed coat and cotyledon were released by alkali-acid and acid-alkali sequential hydrolysis, respectively. Five kidney beans common in extreme Northeast China were selected to investigate the total contents and antioxidant activities of phenols and relevant color attributes. Under the optimized extractions, their TPC and TFC of phenols were relatively high among beans, which showed a significantly positive correlation with antioxidant capacities in vitro. However, phenolic distributions in whole seeds were obviously different between red and white seeds. Moreover, these phenolic indicators were closely associated with seed color. In general, all the phenolic values in red seeds were markedly higher than those in white seeds. Brightness and yellowness could preliminarily anticipate TPC, TFC, and antioxidant activities, and redness depended on TAC. However, redness and spots had no significant effect on the phenolic attributes (except TAC) in red seeds. Among the five kidney beans, the spotted red ‘Yikeshu’ had the highest total content and antioxidant capacity of phenols. A total of 85 phenolic compounds were separated and identified in ‘Yikeshu’ seed with UHPLC-QE-MS. The dominant phenolics included 2 phenolic acids and 10 flavonoids. In addition, the characteristic phenolics in different states and parts of the seed were screened. To our knowledge, this is the first comprehensive study to examine free and bound phenols in the seed coat and cotyledon of kidney beans cultivated in a cold region. The results may help us expand the utilization of kidney beans as a nutritional ingredient in the food industry.

## Figures and Tables

**Table 1:** Appearance and color attributes of kidney bean seeds.

Samples		British Red	Japanese Red	Yikeshu	Zhuhong	Sark
Appearance		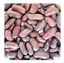	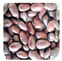	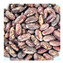	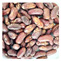	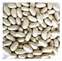
Fitting graph		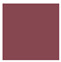	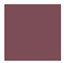	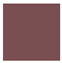	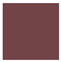	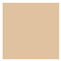
Color attributes	L*	38.45 ± 2.06 b	37.25 ± 1.23 bc	38.76 ± 1.73 b	34.57 ± 2.12 c	80.50 ± 2.15 a
	a*	28.49 ± 1.13 a	21.51 ± 1.31 b	18.77 ± 2.64 b	20.77 ± 1.82 b	7.74 ± 0.98 c
	b*	6.88 ± 0.61 b	3.61 ± 0.20 c	6.09 ± 0.23 b	7.32 ± 0.90 b	21.68 ± 0.96 a
	C*	29.00 ± 0.69 a	21.80 ± 1.28 b	19.95 ± 2.12 b	22.01 ± 2.00 b	22.71 ± 1.55 b
	H*	4.06 ± 0.54 b	5.90 ± 0.41 a	2.97 ± 0.31 c	2.72 ± 0.47 c	−2.82 ± 0.49 d

Different lowercase letters in the same row were statistically significant at *p* < 0.05 (Tukey’s test).

## Data Availability

The original contributions presented in the study are included in the article/Supplementary Materials, further inquiries can be directed to the corresponding author.

## Supplementary Materials

The following supporting information can be downloaded at: https://www.mdpi.com/article/10.3390/foods13111704/s1. Figure S1: Effect of different variables on TPC content for the cotyledon of ‘Yikeshu’ (a) Ethanol Volume Fraction (%), (b) Solid-liquid Ratio, (c) Ultrasonic Power (W), (d) Time (min), (e) Times. Different letters on top of the columns were statistically significant at *p* < 0.05 (Tukey’s test). Table S1: Central composite design and results of TPC of the cotyledon of ‘Yikeshu’ obtained by ultrasound-assisted extractions; Table S2: Qualitative results of free and bound phenolics in the seed coat and cotyledon of ‘Yikeshu’ kidney bean; Figure S2: correlation of the seed coat color in seed. * Correlation was significant at the *p* < 0.05 level. ** The correlation was significant at the *p* < 0.01 level.
