# Supplementary material for: Extractions, Contents, Antioxidant Activities and Compositions of Free and Bound Phenols from Kidney Bean Seeds Represented by ‘Yikeshu’ Cultivar in Cold Region
Source: Foods. 2024 May 29;13(11):1704. doi: 10.3390/foods13111704 (PMC11171797; doi:10.3390/foods13111704)
Supplement: Supplementary file 1 [file foods-13-01704-s001.zip › foods-2982873-supplementary.pdf]

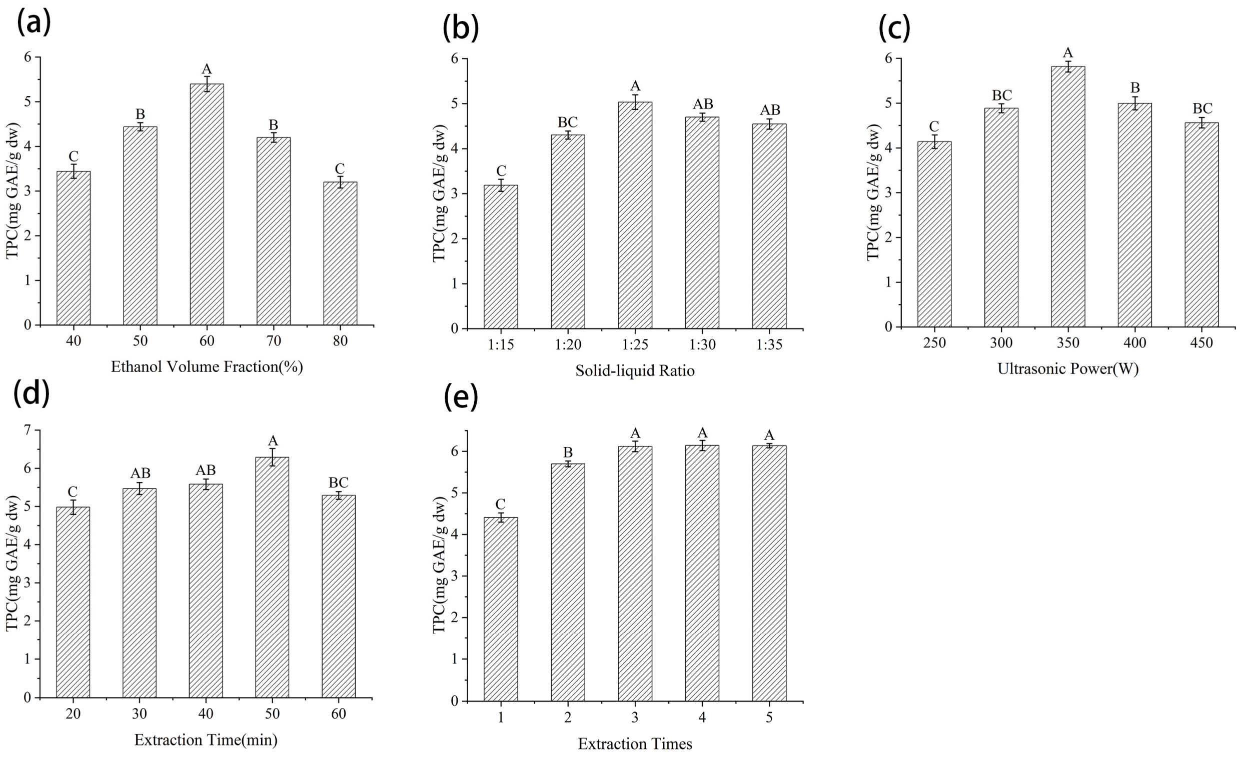

Supplementary Figure S1 Effect of different variables on TPC of free phenol for the cotyledon of 'Yikeshu' (a) Ethanol Volume Fraction (%), (b) Solid-liquid Ratio, (c) Ultrasonic Power (W), (d) Extraction Time (min), (e) Extraction Times. Different letters on top of the columns were statistically significant at $p < 0.05$ (Tukey's test). Abbreviation: TPC- Total phenol content.

Supplementary Table S1 Central composite design and results for cotyledon of ‘Yikeshu’ obtained by ultrasound-assisted extractions.

Experiment	Factors				TPC (mg GAE/g dw)
	Ethanol Volume Fraction (%)	Solid-liquid Ratio	Ultrasonic Power (W)	Extraction Time (min)	
1	-1(50)	0(1:25)	0(350)	1(60)	5.28
2	0(60)	1(1:30)	0	-1(40)	5.33
3	0	1	-1(300)	0(50)	5.77
4	1(70)	-1(1:20)	0	0	5.41
5	0	0	-1	1	5.55
6	-1	0	-1	0	5.28
7	0	1	0	1	5.65
8	-1	0	1(400)	0	5.54
9	0	-1	1	0	5.69
10	0	0	0	0	6.07
11	1	0	0	-1	5.67
12	1	1	0	0	5.87
13	0	1	1	0	5.88
14	0	0	1	1	5.61
15	1	0	0	1	5.42
16	1	0	1	0	5.70
17	0	0	0	0	6.02
18	0	-1	0	-1	5.17
19	0	0	0	0	5.99
20	-1	1	0	0	5.59
21	0	-1	0	1	5.39
22	1	0	-1	0	5.59
23	0	0	-1	-1	5.39
24	-1	0	0	-1	4.88
25	0	-1	-1	0	5.34
26	0	0	0	0	6.07
27	0	0	1	-1	5.86
28	-1	-1	0	0	5.18
29	0	0	0	0	5.98

TPC- Total phenol content.

Supplementary Table S2 Data of free and bound phenolics in seed coat and cotyledon of ‘Yikeshu’ kidney bean by UPLC-QE-MS

Number	Compounds	Retention Time (min)	Mass to charge ratio (m/z)	SF	CF	SBC	SB-AC	CAC	CA-BC	Total
	Phenolic acids									
1	4-O-Caffeoylshikimic acid	3.72	337.09	7.2761E+05	-	1.5309E+06	4.3045E+04	-	1.6034E+05	2.4619E+06
2	Prenyl cis-caffeate	6.04	249.11	3.1769E+05	1.0887E+06	1.8125E+07	4.8333E+06	2.8891E+06	1.3837E+06	2.8637E+07
3	Cafestol	9.28	317.21	8.5911E+04	7.0826E+05	1.1679E+06	-	4.7118E+05	1.6271E+06	4.0603E+06
4	Vinyl caffeate	4.98	207.07	2.6787E+07	3.0119E+07	5.2807E+05	4.0241E+05	6.3148E+06	1.4490E+06	6.5600E+07
5	Ferulic acid	3.06	195.07	4.9313E+06	1.2656E+08	4.2199E+06	4.3940E+06	3.7400E+06	4.1978E+06	1.4804E+08
6	Sinapine	4.59	310.16	1.3070E+05	5.1870E+06	1.8645E+04	1.4377E+04	4.7259E+04	1.7986E+05	5.5778E+06
7	Sinapic acid	3.50	225.08	2.5149E+06	4.5801E+07	7.1762E+05	7.2423E+05	1.8770E+05	5.5461E+04	5.0001E+07
8	Cinnamaldehyde	8.16	133.06	3.0679E+06	3.5782E+06	2.8561E+06	3.3775E+06	1.7828E+06	1.2768E+06	1.5939E+07
9	Ethyl trans-p-methoxycinnamate	8.52	291.20	2.2846E+06	6.2187E+05	-	-	6.6721E+05	7.9854E+05	4.3722E+06
10	Linalyl cinnamate	4.83	285.18	1.5537E+05	1.3901E+07	-	-	-	-	1.4056E+07
11	4-Hydroxycinnamic acid	1.49	165.05	5.4580E+07	5.7454E+08	1.2776E+07	1.2269E+07	1.0311E+07	2.5610E+07	6.9009E+08
12	Coumaric acid	3.74	165.05	9.5246E+06	1.1945E+07	7.8505E+06	8.9376E+06	8.4048E+06	8.6949E+06	5.5358E+07
13	N1,N10-Diferuloylspermidine	7.94	498.26	3.1693E+06	3.7996E+06	8.4148E+04	1.6203E+04	2.6763E+06	9.5280E+05	1.0698E+07
14	Propyl cinnamate	4.71	191.11	8.9486E+05	2.8118E+06	3.8505E+05	-	1.8841E+05	4.7814E+05	4.7583E+06
15	Heptyl cinnamate	5.55	247.17	2.3239E+06	2.0175E+06	2.3031E+05	1.6206E+05	4.1977E+04	1.3687E+05	4.9126E+06
16	N-(p-Hydroxyphenyl)ethyl p-hydroxycinnamide	5.77	284.13	6.3008E+04	2.5580E+06	2.4616E+04	1.7137E+04	3.7082E+07	-	3.9745E+07
17	Diferuloylputrescine	5.80	441.20	1.8535E+06	3.2746E+07	4.0236E+04	-	2.9199E+04	-	3.4669E+07
18	Methyl cinnamate	4.17	163.08	9.7816E+05	4.0020E+07	-	2.5142E+05	5.3815E+05	6.2555E+05	4.2413E+07
	Flavonols									

Supplementary Table S2 (continued)

Number	Compounds	Retention Time (min)	Mass to charge ratio (m/z)	SF	CF	SBC	SB-AC	CAC	CA-BC	Total
19	2-(4-Hydroxyphenyl)-3,6-dimethoxy-8,8-dimethyl-4H,8H-benzo[1,2-b:3,4-b']dipyran-4-one	4.75	381.13	4.9178E+04	2.0495E+07	3.5519E+05	3.1969E+04	9.5631E+04	-	2.1027E+07
20	5'-Hydroxy-3',4',7-trimethoxyflavan	7.84	317.14	3.5238E+05	1.3937E+06	1.9075E+05	2.6366E+05	5.3918E+06	7.1350E+06	1.4727E+07
21	Patuletin	2.73	333.06	-	2.0032E+06	2.6880E+05	-	3.3867E+05	-	2.6107E+06
22	Kaempferol	6.59	287.05	2.9923E+07	2.5731E+06	2.6036E+05	1.7139E+05	4.0228E+05	5.6511E+05	3.3895E+07
23	Quercetin	4.68	303.05	4.1930E+07	6.2664E+07	5.5866E+05	2.8457E+05	-	-	1.0544E+08
24	Kaempferide	5.43	301.07	2.2411E+07	3.0127E+06	3.6105E+05	2.6469E+05	1.6455E+07	1.5204E+05	4.2656E+07
25	Biorobin	5.03	595.17	8.1538E+05	6.0007E+06	-	-	-	-	6.8161E+06
26	Isoquercitrin 4''-rhamnoside	4.81	611.16	1.3983E+06	9.6591E+06	-	-	-	-	1.1057E+07
27	Isoquercitrin	4.97	465.10	3.0065E+07	4.6411E+06	1.5831E+05	1.1492E+04	2.5513E+07	-	6.0389E+07
28	Astragalin	5.20	449.11	1.8143E+08	2.2789E+07	7.1697E+05	1.4201E+04	1.3441E+06	-	2.0630E+08
	Flavones									
29	Isorhamnetin	5.43	317.07	2.9304E+06	5.1812E+05	8.3479E+04	4.7781E+04	4.2067E+05	-	4.0005E+06
30	Luteolin 7-glucoside	4.69	449.11	7.6748E+06	4.2800E+06	1.5091E+05	2.9660E+04	3.5546E+04	-	1.2171E+07
31	Luteolin 7-galactoside	3.87	449.11	1.6417E+08	2.4234E+06	2.3564E+05	-	2.3756E+05	-	1.6707E+08
32	Tangeritin	8.07	373.12	5.1840E+06	9.5756E+06	1.6148E+06	3.6610E+06	1.3272E+06	3.1087E+06	2.4471E+07
33	Apiin	3.81	565.16	5.2339E+06	3.2356E+07	-	-	-	-	3.7589E+07
34	Graveobioside B	4.48	595.17	1.0457E+07	3.6330E+05	-	-	-	-	1.0821E+07
35	Luteolin	6.04	287.05	2.8856E+07	8.5362E+06	7.0703E+05	2.4001E+05	4.4919E+06	1.1207E+05	4.2944E+07
36	5,7-Dihydroxyflavone	5.19	255.06	6.5168E+06	4.2521E+06	3.6703E+05	1.2837E+05	1.0933E+07	2.2994E+06	2.4497E+07
37	Norizalpinin	8.81	271.06	2.2965E+06	-	-	-	6.0712E+04	7.3003E+05	3.0873E+06
38	Nobiletin	7.59	403.14	1.7230E+07	1.7851E+07	5.5769E+06	5.1051E+05	1.8877E+06	3.2823E+06	4.6338E+07
39	Eriodictyol 7-(6-galloylglucoside)	3.89	603.13	4.2462E+05	1.4725E+07	-	-	-	-	1.5150E+07
40	Dihydromorelloflavone	5.79	559.12	8.8615E+06	2.0684E+05	-	-	1.7109E+05	-	9.2394E+06
41	Eriodictyol	4.54	289.07	1.3282E+08	4.8556E+07	3.0766E+07	1.5481E+05	1.0308E+07	2.0863E+06	2.2469E+08

Supplementary Table S2 (continued)

Number	Compounds	Retention Time (min)	Mass to charge ratio (m/z)	SF	CF	SBC	SB-AC	CAC	CA-BC	Total
42	4',5,6,7,8-Pentahydroxy-3'-methoxyflavone	1.97	333.06	6.2205E+04	1.3959E+08	-	-	8.4719E+05	1.6523E+04	1.4052E+08
43	Casticin	5.32	375.11	-	4.4671E+04	6.1934E+05	-	2.1226E+05	-	8.7627E+05
44	2'-Hydroxy-3,4',5',7,8-pentamethoxyflavone	7.64	389.12	2.8836E+05	2.1040E+05	-	-	6.3354E+06	-	6.8342E+06
45	Desmosflavone	0.85	297.11	1.9162E+07	-	-	-	5.2454E+04	1.7627E+04	1.9232E+07
46	2-(3,4-dihydroxyphenyl)-5,7-dihydroxy-6-[3,4,5-trihydroxy-6-(hydroxymethyl)oxan-2-yl]-4H-chromen-4-one	3.93	471.09	1.9627E+06	9.1808E+07	5.9364E+04	-	1.7041E+05	-	9.4001E+07
	Flavanols									
47	3-(4-Hydroxybenzoyl)epicatechin	7.53	411.11	-	-	-	1.9096E+04	4.5936E+06	-	4.6126E+06
48	(-)-Epigallocatechin 3-(4-methyl-gallate)	3.88	473.11	4.3540E+06	6.0760E+06	1.9354E+05	-	8.6668E+04	-	1.0710E+07
49	(+)-Gallocatechin	2.04	307.08	1.1811E+05	2.1641E+07	4.2336E+04	-	3.6094E+06	-	2.5411E+07
50	(-)-Epiafzelechin	4.87	275.09	2.4845E+07	6.2098E+06	2.7788E+06	4.0330E+05	3.0302E+05	8.4725E+05	3.5387E+07
51	Catechin	3.75	291.09	2.3465E+08	5.1817E+08	1.9742E+06	9.3129E+04	6.7929E+05	1.6543E+05	7.5573E+08
52	Epicatechin 3-glucoside	4.05	453.14	6.1172E+06	1.3961E+07	7.3705E+04	1.2955E+04	5.8430E+05	2.2789E+04	2.0772E+07
53	Epicatechin	3.05	291.08	4.5067E+06	1.2167E+08	1.5316E+06	1.6305E+05	-	2.0212E+04	1.2789E+08
54	4'-O-methyl-(-)-epicatechin-3'-O-beta-glucuronide	4.10	495.15	8.1528E+05	-	8.9832E+05	-	5.7730E+04	-	1.7713E+06
	Flavanones									
55	2,4',5,7-Tetrahydroxyflavanone	5.17	289.07	6.3257E+06	1.7349E+07	5.0588E+05	3.5544E+05	7.1940E+06	1.2425E+05	3.1855E+07
56	Sinensetin	7.22	373.13	2.3152E+06	8.1905E+06	3.9172E+05	5.6922E+04	7.3232E+05	8.3350E+05	1.2520E+07
57	Quercetin 3-(6''-malonyl-glucoside)	3.37	301.07	5.7212E+06	8.0798E+07	2.6535E+04	-	3.7114E+05	-	8.6917E+07
58	3,5-Dihydroxy-6,7-methylenedioxyflavanone	4.33	437.11	3.1135E+05	2.3707E+06	1.2988E+06	4.4602E+05	1.9680E+07	1.3968E+06	2.5504E+07
59	5,7,3'-Trihydroxy-4'-methoxyflavanone	4.62	303.09	-	3.6534E+07	6.0689E+04	-	7.3535E+06	2.7894E+06	4.6738E+07

Supplementary Table S2 (continued)

Number	Compounds	Retention Time (min)	Mass to charge ratio (m/z)	SF	CF	SBC	SB-AC	CAC	CA-BC	Total
60	Aromadendrin	3.76	289.07	1.0125E+07	4.0850E+07	1.0538E+06	1.6893E+06	4.4015E+07	6.3305E+05	9.8366E+07
61	Hesperetin 7-glucoside	4.35	465.14	4.6171E+05	8.0987E+05	1.8422E+04	-	-	-	1.2900E+06
62	Blumeatin	4.15	303.09	4.4383E+06	9.4139E+06	-	2.5387E+04	1.6729E+06	2.7687E+06	1.8319E+07
63	Agamanone	4.29	361.09	3.4483E+07	1.6277E+07	3.0440E+05	3.7225E+04	3.2372E+06	1.0636E+05	5.4445E+07
	Isoflavones									
64	Daidzein	5.91	255.06	1.4270E+07	3.7656E+07	1.8276E+05	1.7449E+05	3.1559E+06	1.3694E+05	5.5576E+07
65	Genistein	6.49	271.06	8.7695E+06	5.6116E+06	3.0923E+05	2.8739E+05	9.0667E+05	1.7353E+06	1.7620E+07
66	Genistin	4.42	433.11	4.9223E+07	3.7436E+06	1.5383E+05	-	2.6737E+05	-	5.3388E+07
67	Glycitein	4.53	285.08	4.9151E+06	4.1219E+06	1.3658E+06	2.7624E+05	1.0078E+06	2.7227E+05	1.1959E+07
68	2'-Hydroxydaidzein	3.76	475.12	5.5292E+06	2.3594E+07	1.2377E+06	1.4291E+06	7.9434E+06	1.0287E+05	3.9836E+07
69	Daidzin	5.42	271.06	1.3497E+06	4.4647E+06	-	1.2945E+04	3.4505E+06	-	9.2779E+06
70	6''-Malonylgenistin	4.66	417.12	2.1874E+05	1.1200E+07	-	-	5.5481E+05	-	1.1974E+07
71	Biochanin A	4.84	519.11	3.8254E+05	4.0932E+07	4.9358E+04	1.2785E+04	2.2046E+06	8.4150E+04	4.3665E+07
72	2',7-Dihydroxy-4',5'-dimethoxyisoflavone	6.10	285.08	1.9652E+05	4.9644E+06	6.0607E+04	1.1901E+04	7.0152E+05	-	5.9350E+06
73	2'-Hydroxygenistein 7-(6''-malonylglucoside)	5.93	315.09	7.1551E+05	2.1816E+07	-	-	-	-	2.2531E+07
74	7-hydroxy-6-methoxy-3-(4-methoxyphenyl)-4H-chromen-4-one	4.23	533.13	3.0025E+06	2.7712E+05	5.8424E+05	6.6627E+05	2.6459E+07	9.2884E+05	3.1918E+07
75	Kievitone	6.70	299.09	1.5927E+06	9.9866E+06	-	-	-	-	1.1579E+07
76	Glabrene	7.55	339.12	4.2413E+04	1.2388E+07	-	-	-	-	1.2430E+07
77	Melilotocarpan B	7.54	323.13	2.6065E+06	1.6906E+06	6.0954E+05	1.1626E+05	7.0149E+06	-	1.2038E+07
78	6''-O-Malonyldaidzin	7.27	341.14	3.2385E+05	1.7856E+07	-	-	-	-	1.8180E+07
79	Isogenistein 7-glucoside	4.51	503.12	8.9622E+08	8.4004E+08	1.2386E+06	3.8069E+04	2.9385E+05	8.8242E+04	1.7379E+09
80	cis-4-Hydroxyequol	6.17	355.12	6.9713E+05	5.2091E+06	2.2794E+05	1.5249E+05	1.3255E+06	-	7.6122E+06
81	5-Deoxykievitone	5.10	355.12	3.4045E+06	1.0120E+08	-	-	1.6560E+05	1.7660E+05	1.0495E+08
	Anthocyanins									

Supplementary Table S2 (continued)

Number	Compounds	Retention Time (min)	Mass to charge ratio (m/z)	SF	CF	SBC	SB-AC	CAC	CA-BC	Total
82	Cyanidin 3-galactoside	4.89	449.11	1.8540E+07	-	1.6945E+05	3.3021E+04	-	-	1.8743E+07
83	Peonidin-3-glucoside	5.23	463.12	-	-	1.1497E+05	-	-	-	1.1497E+05
84	Pelargonidin 3-galactoside	5.55	433.11	1.0132E+06	-	-	-	-	-	1.0132E+06
85	Cyanidin 3-glucoside	4.19	449.11	2.7854E+08	-	3.0111E+05	6.3721E+04	-	-	2.7891E+08

Peak area of each phenolic compounds identified in each sample were shown. "-" indicates no detection.

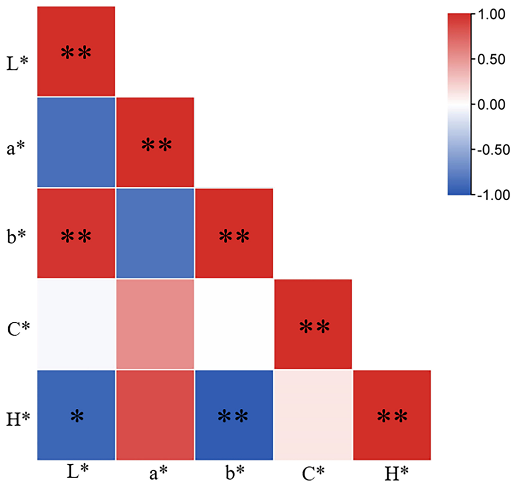

Supplementary Figure S2 Correlation the seed coat color in seed. * Correlation was significant at the p<0.05 level. ** Correlation was significant at the p<0.01 level.
